# Emotion dysregulation, impulsivity and anger rumination in borderline personality disorder: the role of amygdala and insula

**DOI:** 10.1007/s00406-023-01597-8

**Published:** 2023-04-22

**Authors:** M. Mitolo, F. D’Adda, S. Evangelisti, L. Pellegrini, L. L. Gramegna, C. Bianchini, L. Talozzi, D. N. Manners, C. Testa, D. Berardi, R. Lodi, M. Menchetti, C. Tonon

**Affiliations:** 1 Department of Medicine and Surgery, University of Parma, Parma, Italy; 2 Functional and Molecular Neuroimaging Unit, IRCCS Istituto Delle Scienze Neurologiche Di Bologna, Via Altura 3, 40139 Bologna, Italy; 3 Department of Mental Health and Substance Abuse, Local Health Trust of Bologna, Bologna, Italy; 4 Department of Biomedical and Neuromotor Sciences, University of Bologna, Bologna, Italy; 5 School of Life and Medical Sciences, University of Hertfordshire, Hatfield, UK; 6 Hertfordshire Partnership University NHS Foundation Trust, Welwyn Garden City, UK; 7 Department of Physics and Astronomy, University of Bologna, Bologna, Italy; 8 IRCCS Istituto Delle Scienze Neurologiche Di Bologna, Bologna, Italy

**Keywords:** Borderline personality disorder, Amygdala, Insula, Emotion dysregulation, Impulsivity, Anger rumination

## Abstract

Borderline Personality Disorder (BPD) is a severe mental disorder, characterized by deficits in emotion regulation, interpersonal dysfunctions, dissociation and impulsivity. Brain abnormalities have been generally explored; however, the specific contribution of different limbic structures to BPD symptomatology is not described. The aim of this study is to cover this gap, exploring functional and structural alterations of amygdala and insula and to highlight their contribution to neuropsychiatric symptoms. Twenty-eight BPD patients (23.7 ± 3.42 years; 6 M/22F) and twenty-eight matched healthy controls underwent a brain MR protocol (1.5 T, including a 3D T1-weighted sequence and resting-state fMRI) and a complete neuropsychiatric assessment. Volumetry, cortical thickness and functional connectivity of amygdala and insula were evaluated, along with correlations with the neuropsychiatric scales. BPD patients showed a lower cortical thickness of the left insula (*p* = 0.027) that negatively correlated with the Anger Rumination Scale (*p* = 0.019; *r* = − 0.450). A focused analysis on female patients showed a significant reduction of right amygdala volumes in BPD (*p* = 0.037), that correlate with Difficulties in Emotion Regulation Scale (*p* = 0.031; *r* =  − 0.415), Beck Depression Inventory (*p* = 0.009; *r* =  − 0.50) and Ruminative Response Scale (*p* = 0.045; *r* =  − 0.389). Reduced functional connectivity was found in BPD between amygdala and frontal pole, precuneus and temporal pole. This functional connectivity alterations correlated with Anger Rumination Scale (*p* = .009; *r* = − 0.491) and Barratt Impulsiveness Scale (*p* = 0.020; *r* = − 0.447). Amygdala and insula are altered in BPD patients, and these two limbic structures are implicated in specific neuropsychiatric symptoms, such as difficulty in emotion regulation, depression, anger and depressive rumination.

## Introduction

Borderline personality disorder (BPD) is a severe mental disorder, characterized by pronounced deficits in emotion regulation, inappropriate outbursts of anger, interpersonal instability, suicidal/self-harming and impulsive behaviors. A neurobiological model of BPD has been proposed including genetic and environmental factors that affect brain development [1]. Previous neuroimaging findings highlight the presence of several alterations in this population, especially in prefrontal cortex, limbic system and their connection; however, little is known about the specific contribution of different limbic structures to BPD symptomatology [2].

Previous studies described smaller volumes in the amygdala and hippocampus in adults with BPD compared to healthy controls [3–5]; however, when comparing BPD patients with major depression (MD) to BPD without MD, Zetzsche and colleagues [6] showed that amygdala volumes in both hemispheres were significantly larger. Results concerning the role of amygdala are not as conclusive as the results regarding the hippocampus, especially in patients with BPD and other comorbidities. Still under debate is the impact of comorbid post-traumatic stress disorder (PTSD) on limbic grey matter volumes in patients with BPD. A meta-analysis by de-Almeida and colleagues [7] showed that amygdala volumes are reduced in patients with BPD and this pattern is confirmed in BPD patients without PTSD, but not in BPD patients with PTSD.

In the last years, a wide number of neuroimaging studies have used functional MRI (fMRI) approaches to investigate dynamic interactions between brain areas during experimental conditions (i.e. task) and resting states in patients with BPD [8]. Previous task fMRI studies demonstrate increased activation of amygdala bilaterally in patients with BPD compared to controls in response to aversive stimuli (e.g. violent scenes) [9], and patients with BPD also show higher left amygdala activation in response to expressions of emotion depicted in the Ekman faces [10]; they tend to project negative attributes onto the Ekman faces, especially the ambiguous “neutral” expression, suggesting abnormal amygdala functionality [11]. Moreover, resting-state fMRI studies showed aberrant functional connectivity in the default mode network, central executive and salience network in BPD patients compared to healthy controls [12].

A stronger coupling between the amygdala and a cluster comprising the orbitofrontal cortex, putamen and insula was also found by Krause-Utz and colleagues [8]. Insula contains multiple subregions and, as described in the review of Nieuwenhuys [13], is implicated in a large number of widely different functions, ranging from pain perception to the processing of social emotions. Previous neuroimaging studies showed, in healthy volunteers, the presence of two major complementary networks involving the ventral-anterior and dorsal-posterior insula: the first is primarily related to limbic regions which play a role in emotional aspects; the latter is more involved in sensorimotor integration [14]. Only a few preliminary studies explored the involvement of insula in BPD patients [15]; however, no evidence described the contribution of the different insula subparts to BPD symptomatology.

The aim of this study was to explore structural and functional alterations of amygdala and different insula subregions in a group of young adults newly diagnosed patients with BPD, shedding light on their different contribution to neuropsychiatric symptoms.

## Methods

### Participants

Twenty-eight patients with BPD (6 M/22F, mean age = 23.7 ± 3.4 years) and twenty-eight matched healthy controls (6 M/22F, mean age = 24.3 ± 2.8 years) were included in this study. Patients were eligible if they were at least 18 and not over 30 years old and met criteria for BPD at the Structured Clinical Interview for DSM-IV-Axis II (SCID-II) [16]. Exclusion criteria for both groups were: presence of neurological disorders or intellectual disabilities, diagnosis of schizophrenia spectrum disorders or of other personality disorder, comorbidity with substance or alcohol use disorders, and continuous use of poly psychopharmacology over the last year. A subgroup of 20 patients underwent pharmacological treatment, specifically antidepressants, mood stabilizers, antipsychotics and/or anxiolytics.

A total of 20 BPD patients showed a moderate level of depression (Beck Depression Inventory, BDI score > 20); however, no patients had comorbidities with ‘major depression’ nor with ‘Post Traumatic Stress Disorder’ (PTSD). The choice to include young adults and exclude cases with comorbidities was made to assess the BPD core, limiting the impact of other clinical factors that often affect the course of the disease and its neurobiology.

The study was approved by the local Ethical Committee (#88866-24/07/2017), and written informed consent was obtained from all participants.

### Neuropsychiatric assessment

All participants underwent an extensive neuropsychiatric assessment that included the following instruments: Difficulties in Emotion Regulation Scale (DERS) [17]; Barratt Impulsiveness Scale-11 (BIS-11) [18]; Self Harm Inventory (SHI) [19]; Ruminative Response Scale (RRS) [20]; Anger Rumination Scale (ARS) [21]. In addition, the Edinburgh Handedness Inventory [22] was also administrate to evaluate the laterality of each participant; moreover, the Beck Depression Inventory (BDI) was also administrated to the BPD group.

### Brain MR acquisition

Patients and healthy controls underwent a brain MR imaging investigation, within a month after the neuropsychiatric assessment, using a 1.5 T GE scanner (Signa HDx 15) with an 8-channel head coil. The MRI protocol included a 3D high-resolution T1-weighted image acquired using a fast spoiled gradient echo (FSPGR) sequence (pure axial orientation, TR/TE/T1 = 12.3/5.2/600 ms, FOV = 256 mm, 1 mm^3^ isotropic resolution) and a 9 min whole brain resting-state fMRI scan acquired with a gradient EPI sequence (pure axial orientation, TR/TE = 3000/40 ms, FOV = 240 mm, 34 slices, 1.875 × 1.875 × 4 mm^3^ resolution, 180 volumes). All participants were instructed to remain in a relaxed wakefulness, keeping their eyes closed without falling asleep.

### Volume and cortical thickness analysis

**Fig. 1:**
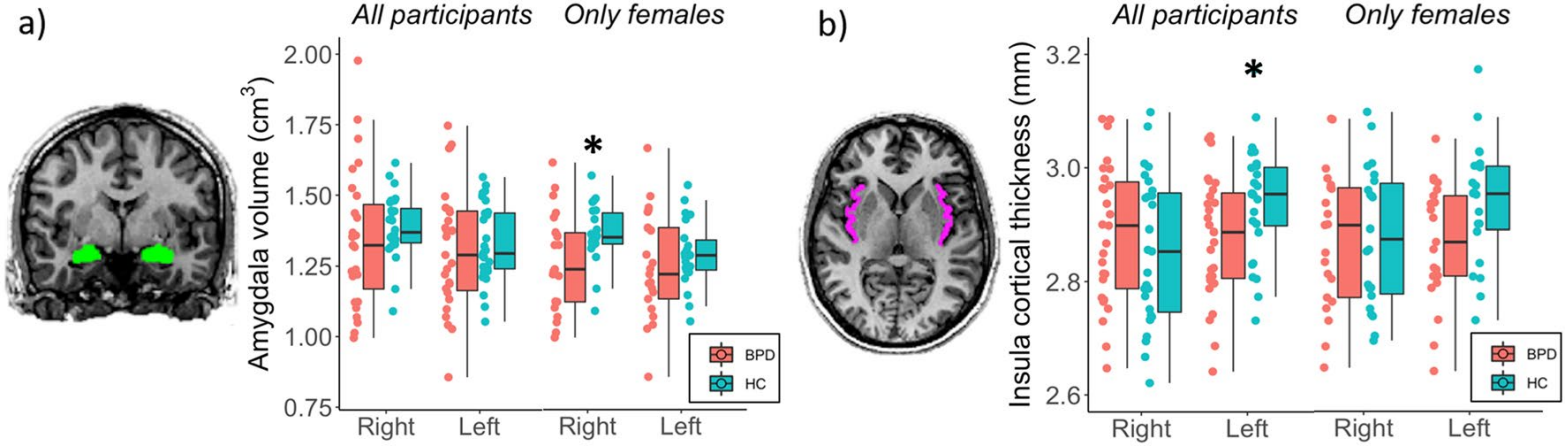
Volume and cortical thickness differences between groups (all participants and only females): **a** amygdala volume; **b** insula cortical thickness. Representative axial and coronal T1-images from a healthy control showing, respectively, amygdala segmentation (**a**) and insular (**b**) (FreeSurfer 6.0 analysis). *p < 0.05

Volumetric and cortical thickness measurements were obtained with FreeSurfer 6.0 image analysis suite (a detailed description of pre-processing and processing of T1-w images is available at http://surfer.nmr.mgh.harvard.edu/) and normalized by the total intracranial volume (TIV, obtained from FreeSurfer) for each participant. Volume was evaluated for both amygdala and insula, while cortical thickness for insula only. Representative segmentations of amygdala and insula are shown in Fig. 1.

**Fig. 2:**
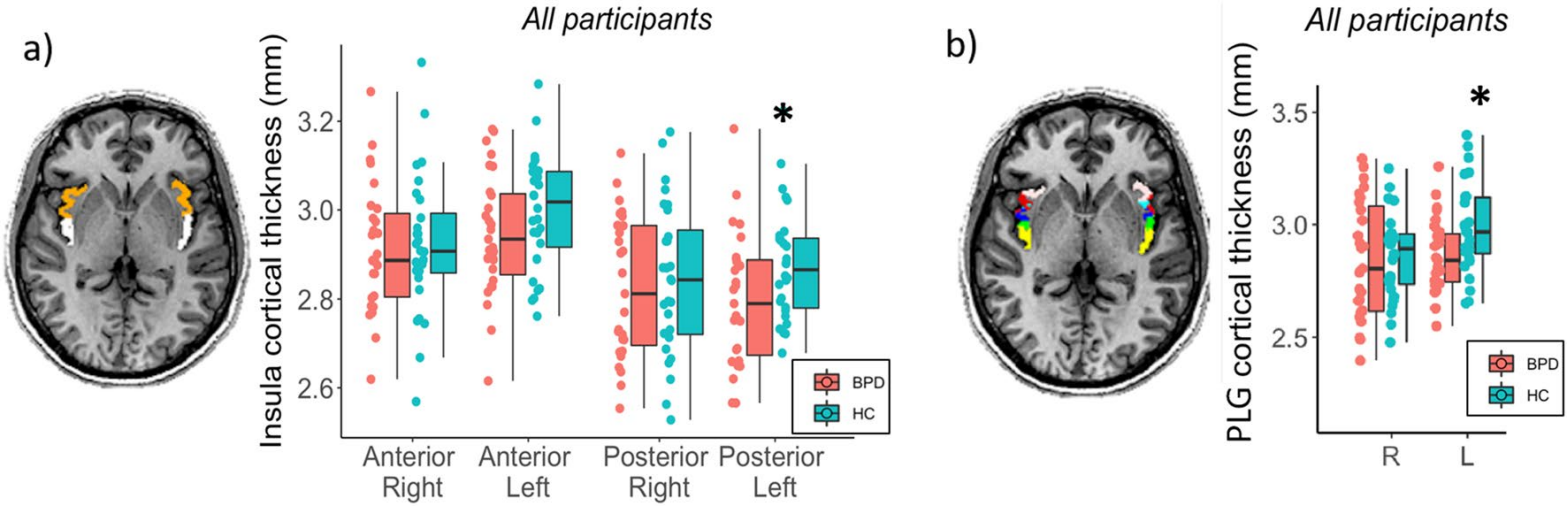
Differences between groups in the cortical thickness of the insula subparts: **a** anterior (orange) and posterior (white) subparts; **b** posterior long gyrus (PLG) (in yellow) obtained from FreeSurfer 6.0 analysis and insula subdivisions (Faillenot et al. 2017); pink: ASG, red: AIC, turquoise: MSG, blue: PSG, green: ALG. *p < 0.05

Moreover, the insular cortex was parcellated into six subregions (posterior long gyrus, PLG; anterior long gyrus, ALG; posterior short gyrus, PSG; middle short gyrus, MSG; anterior short gyrus, ASG; anterior inferior cortex, AIC) as described in the study of Faillenot and colleagues [23], by aligning the subdivision performed on the MNI template to each insula segmentation on the T1-w image. A representative parcellation of insula is shown in Fig. 2.

To explore which specific subregions of the insular cortex were potentially impaired, insula was examined not only as a whole, but also considering the anterior and posterior portions separately (respectively, made of AIC, PSG, MSG, ASG subregions, and ALG, PLG subregions); then, each subregion was also examined individually.

### Resting-state fMRI analysis

Data pre-processing and analysis were conducted using FSL (https://fsl.fmrib.ox.ac.uk/fsl/fslwiki/). Functional images were corrected for head-motion (MCFLIRT) [24]; spatial smoothing (gaussian kernel FWHM = 5 mm) and high-pass temporal filter (cut-off 100 s) were applied.

Functional images were linearly (FLIRT, with BBR method, Boundary Based Registration) [25] registered to the structural T1-w image, and this was non-linearly warped to the MNI template (FNIRT) [26] with a subsequent resample to 2 × 2 × 2 mm^3^.

Single-subject Independent Component Analysis was performed using a probabilistic approach and implemented in MELODIC (multivariate exploratory linear optimized decomposition into Independent components 3.14 FSL tool) with automatically estimated number of components. The obtained components were manually classified between signal and noise, based on knowledge of RSN patterns and of typical artifact characteristics, and data denoising was then performed by regressing out the noise signals [27, 28].

Resting-state data were then analyzed with a seed-based approach, focusing on the amygdala (right and left separately) and the insula (right and left, as a whole, for the posterior and anterior portions separately, and for the PLG part) by aligning the regions of interest used for volumetric analysis to the functional space.

With a dual regression approach between each seed and each subject’s 4D functional dataset, subject-specific spatial maps of functional connectivity were obtained. These maps were aligned to the MNI space and then entered the group-level statistical comparison.

### Statistical analysis

As for clinical and MR volumetric data, parametric t tests were used for group comparisons. The significance threshold was set to *p* < 0.05. Regarding resting-state fMRI data, voxelwise group differences were evaluated with permutation testing (FSL randomize) [29], with 5000 permutations. Age and sex were added as confounding regressors. Statistical significance was set at *p* < 0.05 FWE-corrected for multiple comparisons with TFCE (Threshold-Free Cluster Enhancement). Parameter estimates were extracted from regions that showed significant group differences for correlations.

Group differences were assessed both with whole samples and with sub-groups including only female participants (*N* = 44). Pearson’s correlations were run in the BPD patients’ group between both structural and functional data with the neuropsychiatric scales.

## Results

### Demographic and neuropsychiatric results

The two groups did not differ in any of the sociodemographic factors investigated, and as expected, the neuropsychiatric assessment showed worse scores in the BPD group compared with healthy controls that obtained scores within the normal range in all scales. As shown in Table 1, our sample of patients presents significant higher scores (*p* < 0.0001) on BPD symptoms domains, measured with specific assessment tools, i.e. DERS for emotion dysregulation, BIS-11 for impulsivity, RRS and ARS for rumination and SHI for self-harm.

**Table 1:** Demographic and clinical features of the study groups

	BPD *N* = 28	Healthy controls *N* = 28	*p* value
Age (mean ± SD, years)	23.7 ± 3.4	24.3 ± 2.8	0.465
Gender (M/F)	6/22	6/22	1
Laterality (R/L)	25/3	25/3	1
DERS	121.78 ± 13.96	65.04 ± 17.73	< 0.0001
BIS -11	74.63 ± 10.64	52.89 ± 8.78	< 0.0001
RRS	62.96 ± 9.03	39.32 ± 12.04	< 0.0001
ARS	34.19 ± 7.85	21.57 ± 7.03	< 0.0001
SHI	8.11 ± 4.03	0.79 ± 1.31	< 0.0001
BDI	25.5 ± 8.54	–	–

Laterality was calculated using Edinburgh Handedness Inventory (Oldfield 1971)

*R* right, *L* left, *DERS* Difficulties in Emotion Regulation Scale, *BIS-11* Barratt Impulsiveness Scale-11, *RRS* Ruminative Response Scale, *ARS* Anger Rumination Scale, *SHI* Self Harm Inventory, *BDI* Beck Depression Inventory

### Volume and cortical thickness results

Comparing all patients against controls, no differences were found in amygdala volumes. A focused analysis run with a sub-sample composed only by females (22 BPD and 22 controls) showed a significant reduction of right amygdala volumes (*p* = 0.037) in the BPD group (mean = 1.27 ± 0.18 cm^3^) compared to healthy controls (mean = 1.36 ± 0.10 cm^3^) (Fig. 1). Instead, BPD patients group showed significant cortical thickness reduction of the left insula (*p* = 0.027) (mean = 2.88 ± 0.11 mm) compared to the healthy control group (mean = 2.94 ± 0.09 mm). No significant differences were found considering only the sub-sample of females.

Moreover, the insular cortex was parcellated in anterior and posterior subregions, and the left posterior part resulted significantly thinner in the BPD group compared to healthy controls (*p* = 0.039). A deeper analysis showed that the left posterior long gyrus (PLG) was the subpart significantly thinner (*p* = 0.019) in the BPD group (mean = 2.87 ± 0.16 mm) compared to healthy controls (mean = 2.98 ± 0.19 mm) (Fig. 2).

### Resting-state fMRI results

**Fig. 3:**
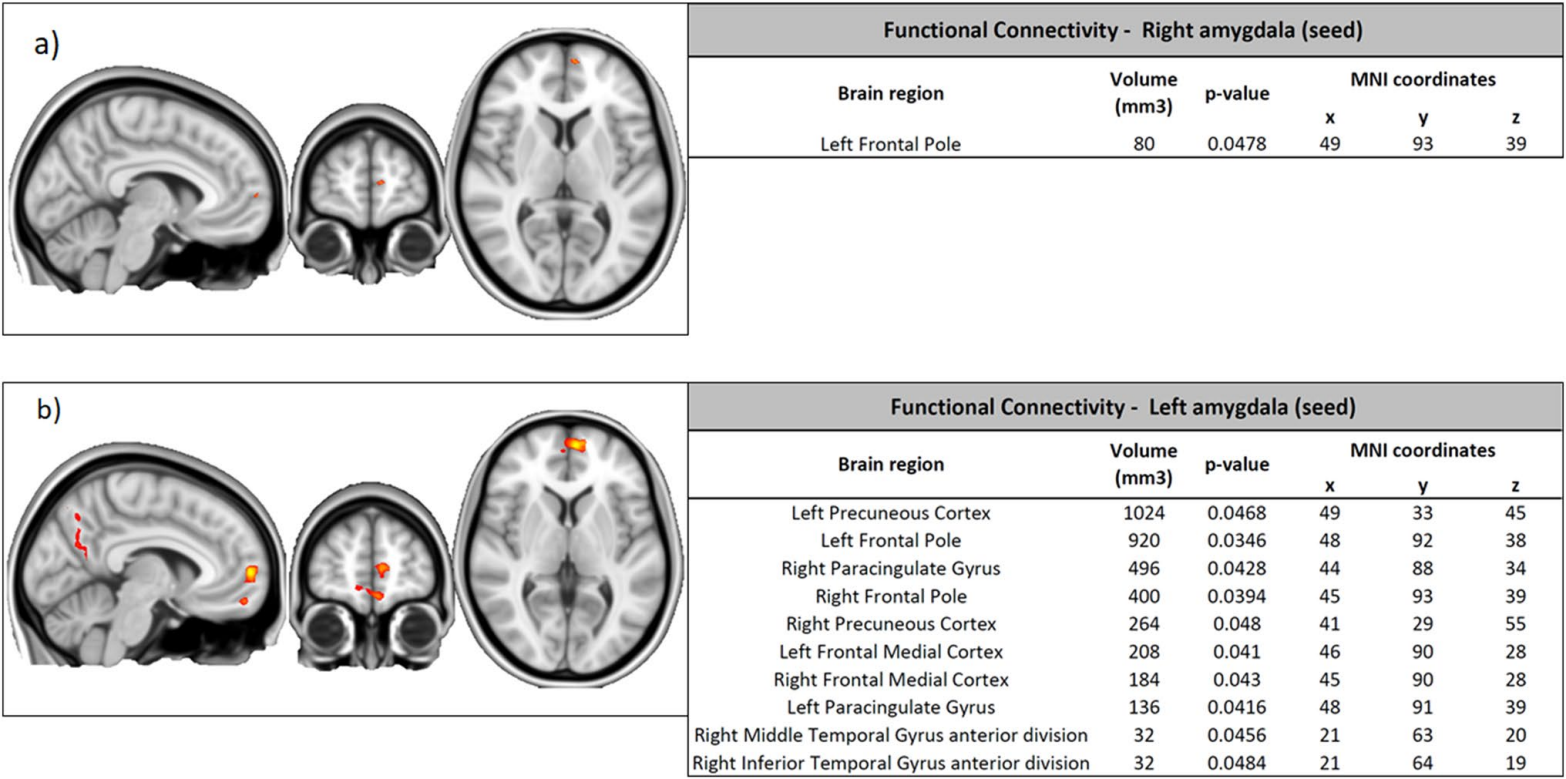
Functional connectivity differences (decreased connectivity) between groups: **a** right amygdala (seed); **b** left amygdala (seed)

The fMRI analysis showed significant reduced functional connectivity in the BPD group compared to healthy controls between the amygdala (right and left) and the frontal pole. In addition, decreased connectivity was also found between amygdala (left) and precuneus and between amygdala (left) and temporal pole (Fig. 3).

When considering only the female sub-groups, reduced functional connectivity in the BPD group was confirmed between right amygdala and the frontal pole; instead, decrease connectivity of the left amygdala was only present at a trend level (*p* < 0.1).

**Fig. 4:**
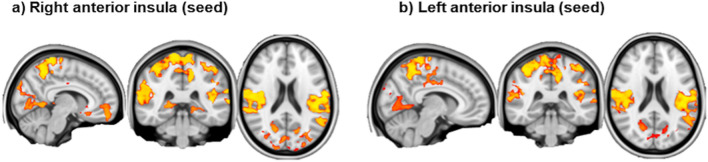
Functional connectivity differences (increased connectivity) between the two female sub-groups: **a** right anterior insula (seed); **b** left anterior insula (seed)

No differences in anterior nor posterior insula functional connectivity were found between patients with BPD and healthy controls when considering the whole sample. A focused analysis run only with the female sub-groups showed significantly higher connectivity in subjects with BPD between anterior insula (right and left) and several brain areas (Fig. 4).

### Correlations analyses

Negative correlations were found between right amygdala volume and two neuropsychiatric scales, specifically, Difficulties in Emotion Regulation Scale (*p* = 0.031; *r* =  − 0.415) and Ruminative Response Scale (*p* = 0.045; *r* =  − 0.389). Moreover, significant negative correlations were also found in the BPD group between cortical thickness of the left insula and Anger Rumination Scale (ARS) (*p* = 0.019; *r* =  − 0.450). Regarding functional connectivity alterations, significant positive correlations were found between reduced left amygdala connectivity and two neuropsychiatric scales, specifically, Anger Rumination Scale (*p* = 0.009; *r* = 0.491) and Barratt Impulsiveness Scale-11 (*p* = 0.020; *r* = 0.447). Instead, correlations between increase left anterior insula connectivity with the neuropsychiatric scales (i.e. Difficulties in Emotion Regulation Scale) were only present at a trend level (*p* = 0.09; *r* =  − 0.37).

## Discussion

The results of this study clearly demonstrate the presence of amygdala and insula structural alterations in a BPD sample, highlighting their association with specific neuropsychiatric symptoms, such as emotion dysregulation, rumination and impulsivity. In addition, reduced functional connectivity between amygdala and different brain areas, mainly frontal areas, was also found in the BPD group compared to healthy controls.

Previous functional brain imaging studies provide an approach in assessing the relationship between prefrontal cortex (PFC) and amygdala function by examining the correlation coefficients between these two structures [30]. Human studies, mainly carried out in healthy subjects, with fMRI and with 18-fluorodeoxyglucose (FDG)-positron emission tomography (PET), showed significant correlation between these two structures; however, the directionality of these correlations was inconsistent. Some studies showed positive correlations between regions of PFC and amygdala [31]; instead, other studies negative correlations [32]. The amygdala has been implicated not only in the processing of negative emotions in general, but also more specifically in the regulation of aggressive behavior. In primates, bilateral ablation of the amygdala leads to increased social affiliation and decreased aggression [33, 34], demonstrating a central role of the amygdala in the processing of aggressive behaviors. Moreover, recent human studies showed that bilateral amygdala radio-frequency ablation improved behavior in patients presenting refractory aggressive behavior [35].

Our data showed in a BPD population the association between functional amygdala alteration and two symptoms related to pathological aggression: impulsivity and anger rumination. The association between impulsivity traits and amygdala in patients with borderline personality disorder has been recently described by Eskander and colleagues [36]; instead, the neural correlates of anger rumination, a poorly studied construct, remain a matter of debate.

In this study, we also found an association between anger rumination and reduced insula cortical thickness, indicating a role played by both limbic structures in this specific trait. These findings contribute to increase the understanding of the neural processes related to the risk of aggressive behavior by specifying that both insula and amygdala are involved in the subjective experience of anger rumination that, as described by Martino and colleagues [37], predicts aggression proneness. As the Emotional Cascades Model (ECM) [38] states that emotional dysregulation is amplified and exacerbated by rumination in a positive feedback loop, which leads to ‘cascades of emotion’ [1], in this study, we showed a strong association between impulsivity and anger rumination, which are both related to limbic structures alterations (i.e. amygdala and insula).

To the best of our knowledge, this is the first study that describes the neural correlates of anger rumination in a BPD population using both structural and functional techniques. In a previous study, Denson and colleagues run an fMRI experiment, with healthy young volunteers, during which participants were insulted and induced to ruminate [39]. Authors found that different neural regions, including insula, have been specifically linked to anger rumination. More recently, an fMRI study with 13 women with BPD, despite limitations related to the small sample size, found increase activation in the insula and in the orbitofrontal cortex in response to negative feedback [40]. As the insula regulates affective distress, and its alteration in patients with BPD is involved in reactivity to social rejection, this result was interpreted as contributing to their greater reactivity to criticism and their tendency to put greater efforts to regulate emotional responses [40]. Beside these preliminary evidence, we found specific association with functional but also structural alteration of amygdala and insula when considering only the female subgroup of patients with BPD, but also when considering the whole sample. The ratio of males/females of BPD patients recruited in this study is consistent with epidemiological studies and with the routine access to community mental health services, where patients are usually referred [41]. Hence, despite the number of studies in borderline personality disorder patients, gender differences related to symptoms and/or associated with specific brain structural and functional alterations have not been adequately addressed in the literature; therefore, results are still inconclusive.

The results of this study clearly demonstrate the presence of amygdala and insula alterations in a BPD population and these two limbic structures seem to be implicated in different neuropsychiatric symptoms. Further studies with larger and homogeneous samples, controlling for gender and comorbidities, are needed to confirm these preliminary results and to provide additional insights about the association between specific neuropsychiatric symptoms and different brain structures, shedding light on the relevance of neuroimaging biomarkers in borderline personality disorder.
